# Barts and The London School of Medicine and Dentistry

**DOI:** 10.1038/s41415-022-5209-3

**Published:** 2022-11-25

**Authors:** Paul Coulthard

**Affiliations:** Professor of Oral and Maxillofacial Surgery and Dean and Director, Institute of Dentistry, Barts and The London School of Medicine and Dentistry, Queen Mary University of London, E1 2AD, UK

## Abstract

The Institute of Dentistry at Barts and The School of Medicine and Dentistry, Queen Mary University of London, has seen much change since 1857 when surgeon dentist, H. J. Barrett, was appointed to The London to oversee the extraction of teeth. However, the mission remains the same: provision of excellent patient care through our education and research to improve the oral health and general health of our local population, but also with global impact.

The Institute of Dentistry is embedded in a fundamentally multidisciplinary environment of the wider university and this is reflected in our research activity. The available complementary skills in cell and molecular biology, microbiology, materials science, chemistry, biophysics, clinical science and population health allows us to undertake basic science research, patient- and population-based research and clinical biometric research.

Our Centre for Teaching and Innovation is a catalyst for educational research, including that related to new technologies and the expansion of e-learning, to ultimately inform local practice and the experience of our students.

## Key points

- The impact of the COVID-19 pandemic on clinical services and therefore education and our research was huge. Fortunately, successful bids to Health Education England for funds to support training recovery enabled catch-up of clinical experience with evening and weekend clinics. We were able to graduate students as usual with the same experience as in pre-COVID-19 times.
- Barts Centre for Squamous Cancer is a new centre of excellence dedicated to improving detection, treatment and quality of life for patients with squamous cancer.
- The Institute of Dentistry is using 42 student virtual reality Simodont dental trainers that apply sophisticated haptic technology with a highly realistic haptic (force) feedback provided through a dental drill handpiece.

## Dental institute historical context

Barts and The London School of Medicine and Dentistry at Queen Mary University of London (QMUL) was formed in 1995 by the merger of the London Hospital Medical College and the Medical College of St Bartholomew's Hospital. The London Hospital Medical College was the first school in the UK to be granted an official charter for medical teaching in 1785. The Medical College of St Bartholomew's Hospital is the oldest remaining hospital in the UK, having been founded in 1123, with medical teaching beginning from that date. The nine-hundredth anniversary of St Bartholomew's Hospital takes place in 2023!^1^

In the mid-nineteenth century, dentists in Britain wanted to increase their standing in society, as did doctors, by developing qualifications. Two major groups were influential: the Odontological Society of London and the rival College of Dentists of England. The members of the Odontological Society were mainly surgeons practising dentistry in London, who wanted dental surgery to be a speciality of surgery. The practitioners of the College of Dentists of England wanted dentistry to be totally separate from surgery.^2^ A power struggle continued between the two groups until the Royal College of Surgeons was granted a new royal charter, allowing the embryonic dental profession to have its first British qualification in 1860 - the Licence in Dental Surgery (LDS) of the Royal College of Surgeons of England. Once the Royal College of Surgeons instituted the LDS, the College of Dentists no longer had a purpose and merged with the Odontological Society.^3^

The London Hospital appointed a surgeon dentist, H. J. Barrett, to oversee the extraction of teeth in 1857. This developed into a dental department within the general hospital and then an Institute of Dentistry was opened in 1911.^4^ The main points of submission to the Royal Commission in 1910 included 'dentistry is a branch of medicine because of the relationship between general and dental disease and the dentist needs knowledge of such related diseases'. There has been a widening separation over time between medicine and dentistry, such that we are now needing to remind both professions of the obvious interplay and to 'put the mouth back into the body'.

By the 1920s, restorative dental materials had changed very little and were to remain unchanged for a good many years. Silver-tin amalgam was an essential mainstay but it was still felt unnecessary to disabuse students of the idea that the mercury could harm patients - the dangers to operator and assistants received no mention. Cavity preparation was firmly based on the precepts of G. V. Black and the doctrine of 'extension for prevention'. Local anaesthesia was rarely used in conservative treatments. Arsenic was routinely used for devitalisation and a crystal of cocaine was recommended for application to an accidental exposure of the pulp.^5^

The University of London had established a Bachelor of Dental Surgery (BDS) degree in 1922 but this conflicted with the LDS and few took the degree examinations until arrangements were made to take both. By 1945, all students took the BDS at the Institute of Dentistry of the London Hospital. World War II and the succeeding years brought a number of new materials which permitted refinement and improvement in a number of technical procedures. The most obvious change, for a dental profession still much concerned with complete dentures, was the complete replacement of the opaque poorly coloured vulcanite by poly-methylmethacrylate, which was translucent and available in a variety of colours.^6^

Planning began in the 1950s for a new Institute of Dentistry building to accommodate a doubling of the student intake and the building on Stepney Way was moved into in 1965. The London Hospital warmly supported the venture of the New Cross Auxiliary Training School set up in 1960 and transferred it to the new Institute of Dentistry. The young women (there were no men) of the auxiliary school undertook restorative work and extractions for school children and oral hygiene instruction and scaling. The British Dental Association and the large proportion of the profession viewed the auxiliaries with deep suspicion. The American positive experience where hygienists first appeared in 1907 failed to carry any conviction with the profession.^7^ Interestingly, we are still debating the employment of hygienists and therapists in 2022, with surprisingly limited understanding by many of their roles in the team!

**Fig. 1:**
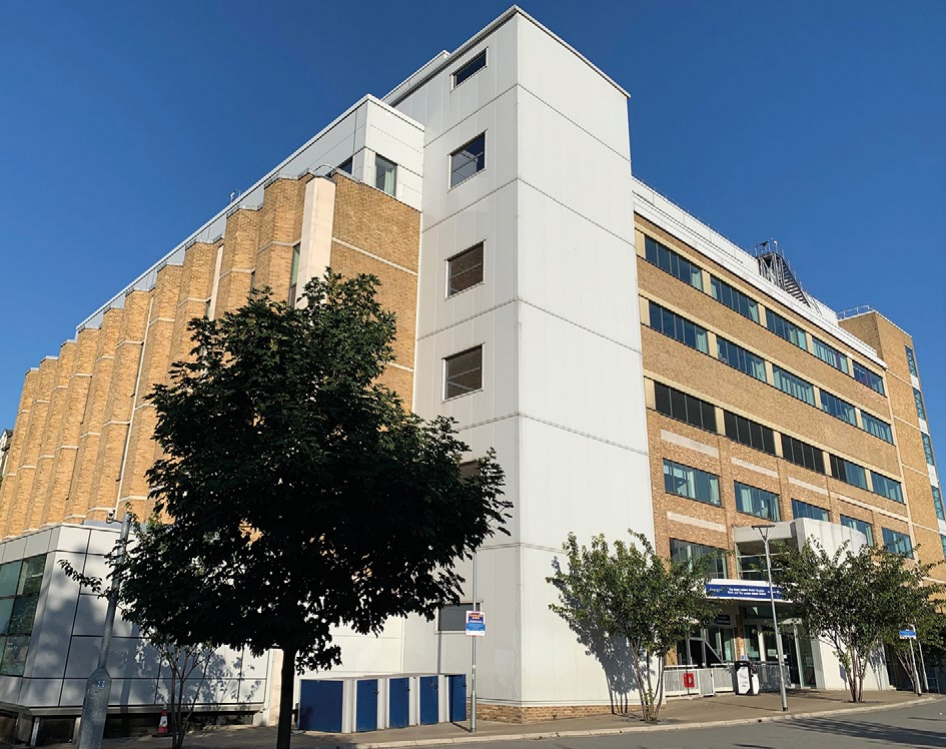
The Royal London Dental Hospital and Institute of Dentistry building

**Fig. 2:**
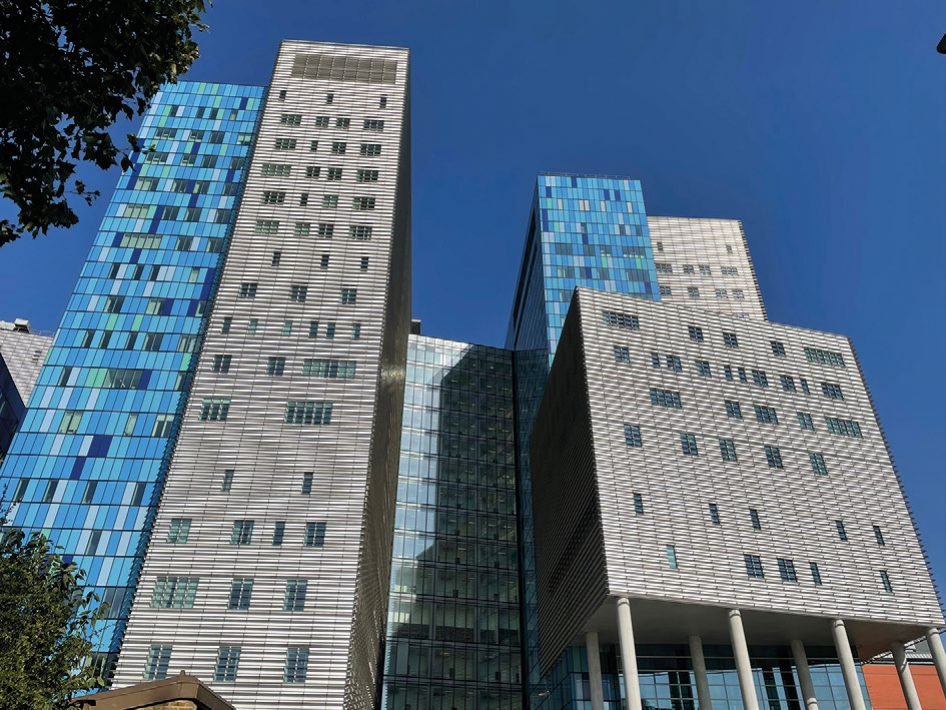
The Royal London Hospital, a hospital of Barts Health NHS Trust

**Fig. 3:**
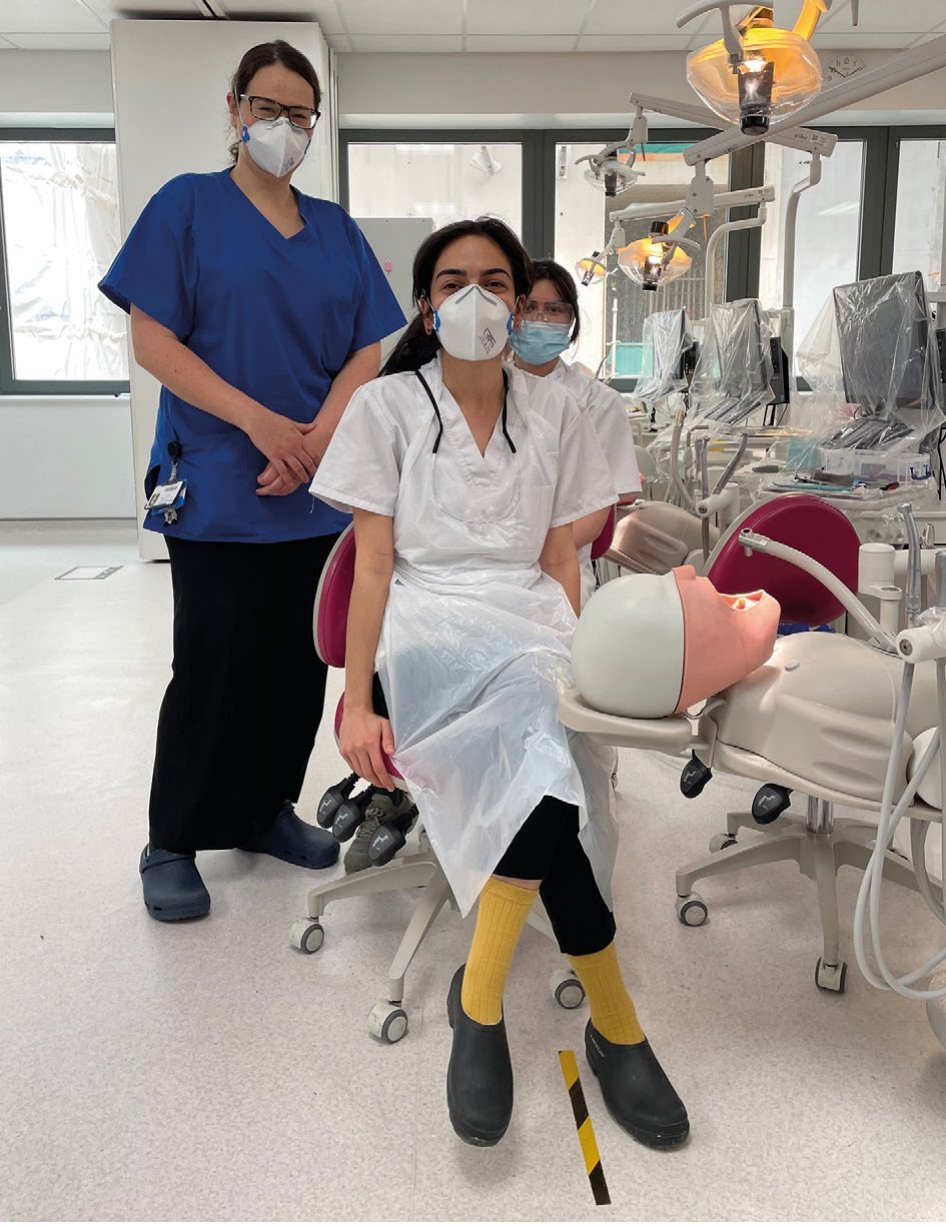
The traditional clinical skills classroom at the Royal London Dental Hospital

A £78 million new Royal London Dental Hospital and Institute of Dentistry building in Whitechapel was completed in 2014, adjacent to the Royal London Hospital building. The building provides excellent, state-of-the-art, clinical, educational and research facilities (Figures 1, 2 and 3). There is a new oral clinical research unit. The Dental Hospital is situated in close proximity to the world-leading Blizard Institute, which fosters a collaborative working environment with colleagues in medicine and allows the Institute of Dentistry to take advantage of QMUL's exceptional translational medicine expertise and facilities.

## Research strategy

In the early 1970s, the education aspirations of the London Hospital were stated. These recognised the improvements in restorative and preventive dentistry and the greater use of fluoridation. It was also noted that there was a rise in the numbers of those of advanced years, the employment of auxiliaries and changing societal factors, such that there was a need to move from basic manual skills to education that would fit dentists to understand the patient as a whole and be competent in case analysis and treatment planning. It was also stated that there should be more emphasis on the scientific base of practice and more research, including research into the educational process itself. This holistic approach is now our continued focus so that our graduates are public health and evidence-based thinkers, as well as practitioners.

The Medical Research Council established the Epidemiology of Dental Studies Unit and a Master of Science (MSc) in Dental Public Health and in Experimental Oral Pathology was instigated. Research areas grew, an external grant income was awarded and Doctor of Philosophy (PhD) studentships blossomed. We now have a suite of postgraduate programmes, including nine MScs and eight Doctor of Clinical Dentistry degrees on offer.

Barts and The London School of Medicine and Dentistry has continued to have a productive research output and as research income, government assessment of research performance and world ranking of departments have become increasingly important to universities, then this is a major mission of the Institute of Dentistry. Dental school research is for the benefit of patients' oral and general health.

Dental school research has become more integrated with wider biomedicine over time and it is recognised that collaboration is key to success. Barts and The London Institute of Dentistry still has strong dental materials research but also develops new materials for maxillofacial and orthopaedic surgical use. The Institute of Dentistry is embedded in a fundamentally multidisciplinary environment of the wider university and this is reflected in our research activity. The available complementary skills in cell and molecular biology, microbiology, materials science, chemistry, biophysics, clinical science and population health, alongside the design of our research spaces, allows us to undertake both basic science research, patient- and population-based research and clinical biometric research. The 40 academic research staff are led by the Dean and work in three research centres: dental public health; oral bioengineering; oral immunology and regenerative medicine, including the oral cancer group. There is a wide range of research undertaken across diverse areas, including Behcet's disease, dental sleep medicine, bioactive glass development, dental imaging, nitric oxide delivery systems for wound dressing and improving the oral health of vulnerable children.

## Barts Centre for squamous cancer

The Barts Centre for Squamous Cancer is a new centre of excellence, dedicated to improving detection, treatment and quality of life for patients with squamous cancer. Squamous cancers are the most frequent human solid tumours and a major cause of mortality. Squamous cancers can form in the mucosa of the oral cavity and oesophagus, the skin, the lungs and the cervix. Recent pioneering Barts research has shown that these tumours have common determinants and this has raised exciting opportunities for new strategies for cancer prevention, detection and treatment. The Dean, Paul Coulthard, Professor of Oral and Maxillofacial Surgery, together with Irene Leigh, Professor of Cellular Molecular Medicine and a dermatologist, set up the Barts Centre for Squamous Cancer in 2021 with over £2.6 million funding from Barts Charity. This is a cross-institute collaborative centre bringing together research groups with diverse expertise from across the School of Medicine and Dentistry to tackle the problem of squamous cancer and drive clinical innovation.

## COVID-19 pandemic

**Fig. 4:**
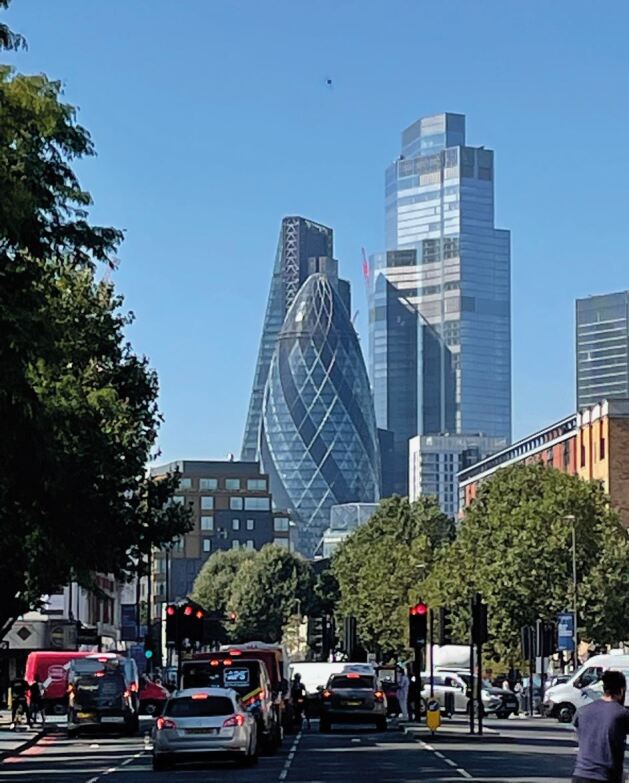
View of the City of London from the Institute of Dentistry

**Fig. 5:**
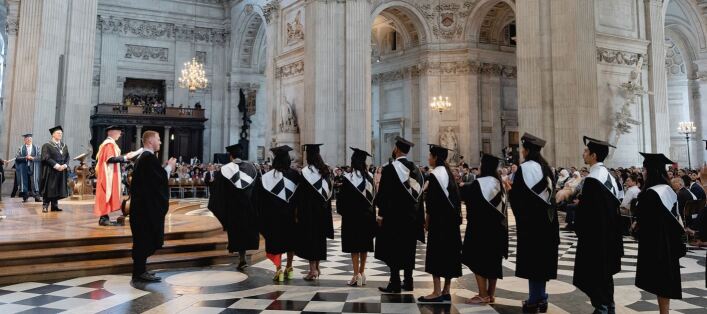
Rites of Passage ceremony at St Paul's Cathedral for dental and medical students

**Fig. 6:**
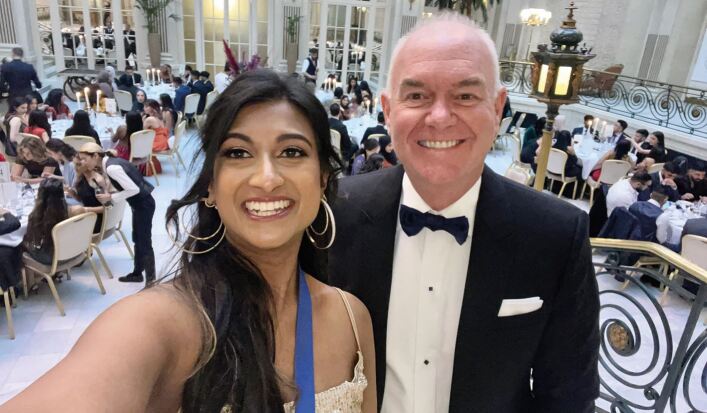
Dean and Institute Director, Professor Paul Coulthard, having a selfie with Barts and The London Dental Society President, Jathursha Suresh, at a dental dinner in 2022 (The Waldorf Hilton, Aldwych, London)

The Institute of Dentistry was hit particularly hard by the COVID-19 pandemic during 2020 and the second wave during early 2021 due to its East London location serving some of the most deprived and unwell people in the country, despite being a short walk to the City of London (Fig. 4). Many clinical, nursing and administrative staff were redeployed to medical duties and the hospital/school building was repurposed for medical care. The impact on clinical services and therefore our undergraduate and postgraduate education and our research was huge. Fortunately, successful bids to Health Education England (HEE) for funds to support undergraduate Bachelor of Dental Surgery and Bachelor of Science training recovery enabled catch-up of clinical experience with evening and weekend clinics. We were able to graduate students as usual with the same experience as in pre-COVID-19 times. Students were, however, anxious during this time and staff were exhausted. The QMUL graduation and the student-organised 'Rites of Passage' ceremony in St Paul's Cathedral (Fig. 5) were particularly joyous. Student balls are also now back (Fig. 6)!

## Dental school funding

The national economy and funding to dental schools has a major impact on development and schools need to take advantage of opportunities. Income comprises that from tuition fees, external research funding and that from HEE. The latter is the most significant income and provides the funding for the clinical training; however, this is paid directly to the partner NHS Hospital Trust rather than the dental school. For some decades, this model has been criticised by the dental deans, who are held responsible by the General Dental Council (GDC) for the graduation of dental professionals fit for registration to practise but with little control of the funding to facilitate it and significant opacity around the spend of this funding within the trusts. Ultimately, this is taxpayers' money that is supposed to be for dental clinical education and so it is good to see that HEE are wanting to see greater transparency and evidence of the spend on dental education.

The A-level grade inflation that followed teacher-assessed grades in 2020 and particularly so in 2021 resulted in a significant opportunity for dental and medical schools. There is a tightly enforced cap on student numbers for dentistry and medicine, supposedly linked to workforce needs. In my opinion, successive governments have been reluctant to pay for the appropriate number of dentists to be trained because it is very expensive and have relied instead on training elsewhere in Europe to make up deficit. Brexit has resulted in huge workforce problems, showing initially in coastal/rural areas, and then everywhere. So, the A-level grade inflation provided an opportunity to contribute to providing more dentists for workforce as there was a temporary lifting of the cap on numbers and a government commitment to funding more students. The Minister of State for Universities wrote to deans of medical and dental schools asking us not to defer students to the next year but urging us to find ways with government financial support to manage our systems and capacity. The Institute of Dentistry at Barts and The London Institute for Dentistry was therefore delighted to take many more students, although, of course, there is much work in managing systems and capacity!

## Dental educational research

The pursuit of educational excellence is a fundamental priority to the Institute and we are developing our research in this area. Our 'Centre for Teaching and Innovation' is a catalyst for educational research, including that related to new technologies and the expansion of e-learning, to ultimately inform local practice and the experience of our students. Our research is to enhance the quality of care and optimise patient safety. We are currently leading work involving all UK dental schools concerning professionalism and fitness to practise in collaboration with our professional regulator, the GDC. The expected future impact is a reduction in GDC fitness to practise cases for UK dental graduates. Continued work around the interface of dental undergraduate education and HEE-funded foundation training will increase the understanding of the interface and increase the effectiveness of foundation training.

## Digital dentistry

Digital dentistry is an emerging diagnostic and therapeutic modality, with significant impact for clinical dentistry.^8^ The Institute of Dentistry and Barts Health Dental Network were awarded £2.3 million in 2022 from Barts Charity for digital transformation. The project includes the modification of the dental-hospital-wide electronic record system to allow detailed dental assessments and extensive data capture linked to wider general hospital services. The electronic data will then be used to undertake interdisciplinary research. The third part of the project will improve clinical learning and training of undergraduates, trainees and postgraduates using virtual reality and haptic stations.

The Institute of Dentistry already has 27 virtual reality Simodont dental trainers and the research grant increases the number to 42. These Simodont dental trainers apply sophisticated haptic technology used in flight simulators, adapted for dental simulation with a highly realistic haptic (force) feedback provided through a dental drill handpiece.^9^^,^^10^ The aim is to improve preparedness of students to treat patients. Exercises provide immediate, objective feedback to the learner, allowing rapid improvement of hand-skills. Teachers can observe and evaluate the work of students either in real time or offline at a later point in time.

Haptic trainers also provide an opportunity to upload patient-specific dental information and images. This will allow a student to practise a particular procedure on a particular tooth of their patient, virtually, before undertaking the same procedure on the actual patient.

## Conclusion

The Institute of Dentistry at Barts and The London School of Medicine and Dentistry of Queen Mary University of London has seen much change since 1857, when surgeon dentist, H. J. Barrett, was appointed to oversee the extraction of teeth. There are many external factors that impact development, such as world wars, the economy and pandemics, but the mission remains the same: provision of excellent patient care through our education and research to improve the oral health and general health of our local population but also with global impact.
